# Between culture and religion: explanatory models of mental health conditions amongst Muslims in Cape Town, South Africa

**DOI:** 10.3389/fpsyg.2026.1910057

**Published:** 2026-09-25

**Authors:** Mariam Salie, Ashraf Kagee

**Affiliations:** Department of Psychology, Stellenbosch University, Stellenbosch, South Africa

**Keywords:** culture, explanatory models, mental health, muslim, religion

## Abstract

**Introduction:**

Mental health remains highly stigmatized across many cultural and religious contexts, including Muslim communities in South Africa (SA), where Muslim mental health is under-researched. With Islam being one of the fastest-growing religions globally, alongside increasing global stressors affecting Muslim populations, there has been a rise in demand for mental healthcare. Recent literature highlights the importance of culturally relevant care, particularly through understanding Muslims’ explanatory models of mental health conditions to ensure responsive and appropriate services. However, existing studies in SA remain limited and dated.

**Methods:**

This study explored the explanatory models of mental health conditions among Muslim psychological service users and members of the general Muslim public. Semi-structured individual interviews were conducted with service users, and focus group discussions were held with public participants. A total of 39 participants (21 service users and 18 public members) were recruited through purposive and snowball sampling. Data were analyzed inductively using reflexive thematic analysis.

**Results:**

Findings highlight diverse explanatory models encompassing beliefs, attitudes, causal explanations, perceptions of severity, and treatment preferences. Mental health was understood through an integrated framework of Islamic teachings, cultural norms, and societal expectations. Faith-based coping strategies, such as prayer, trust in Allah, and religious practices, were widely endorsed. However, stigma remained prominent, with mental health struggles often described as “not spoken about.” This silence was linked to fear of social judgement, misconceptions, and limited mental health literacy.

**Discussion:**

Findings demonstrated the multifactorial, complex nature of explanatory models and the influence of broader contextual factors on them. These models are continually negotiated in experiences of psychological distress. Experiences of isolation and delayed help-seeking were emphasised in the community, demonstrating that explanatory models influences help-seeking behavior.

## Introduction

There is a growing recognition in global mental health studies of the need to move beyond purely biomedical perspectives when understanding mental health conditions. These studies, like (Alharbi et al., 2021; Al-Maamari et al., 2025; Awaad et al., 2019; Ayinde et al., 2021), increasingly emphasise the importance of understanding explanatory models of mental health conditions. Mental health conditions is used in this paper as an umbrella term and includes all categories of psychological and psychosocial disorders, beyond traditional biomedical frameworks. Understanding explanatory models are important to mitigate barriers for accessing mental health care, and to support practitioners in developing cultural sensitivity in their clinical practice. Understanding cultural expressions of mental health challenges can reduce the risk of pathologising culturally diverse, non-Western expressions of distress.

Religion typically plays an important role in the lives of observant Muslims (Awaad et al., 2019; Laher, 2014), including how they conceptualise mental health conditions (Alharbi et al., 2021; Al-Maamari et al., 2025; Awaad et al., 2019). Studies have found that concepts such as divine punishment, lack of faith or religiosity, and supernatural explanations, such as black magic, evil eye and spirit possession, have all been implicated in Muslims’ explanatory models of mental health conditions (Bulbulia and Laher, 2013; Haque, 2004; Mitha, 2020). However, these explanatory models are further influenced by geographical location, sociopolitical factors and cultural beliefs (Bhugra et al., 2021). Thus, beliefs and perceptions about mental health will vary across different Muslim communities (Alharbi et al., 2021). These differing beliefs render the interplay of religion and culture a challenge in understanding Muslim mental health (Awaad et al., 2019). While numerous definitions of culture exist; for the purpose of this paper I draw on the definition by Matsumoto and Juang (2023) which defines culture as *“the set of attitudes, values, beliefs, and behaviors shared by a group of people, but different for each individual, communicated from one generation to the next*.” Cultural practices thus function on a set of underlying assumptions shared by communities (Spencer-Oatey, 2012) and ultimately influence behavior. Of course, culture is influenced by other factors too, including religion (Hofstede, 1991). There is a need to understand cultural explanatory models of mental health conditions to build collaborative care (Alharbi et al., 2021; Al-Maamari et al., 2025; Awaad et al., 2019; Ayinde et al., 2021).

The term *explanatory model* was coined by medical anthropologist Arthur Kleinman (Kleinman, 1978a, 1978b) and highlights the integration of his anthropological and clinical approach to understanding illness. Explanatory model refers to an understanding of how patients perceive their illness, their ideas about how the illness developed, and beliefs about its course and treatment. He argued that understanding a patient’s explanatory model facilitated patient-centered care and improved treatment outcomes (Kleinman, 1980). Understanding the patient’s explanatory model would thus highlight any informal help-seeking behavior, and other cultural, social, and structural factors that may hinder formal treatment or create barriers to treatment adherence (Apers et al., 2023; Bokhour et al., 2012). Ultimately, the goal is to develop culturally sensitive treatment interventions that are patient-centered, responsive to patient needs and aligned with patient explanatory models (Apers et al., 2023; Arabiat et al., 2021).

Muslims believe and submit to the will of *Allah* (God) and typically operate on three significant Islamic principles, a monotheistic belief system, humankind as vicegerents of God, all equal, blessed with resources as a trust, and justice (Abdullah and Nadvi, 2011). Muslims believe in the Qur’an as the word of God, and follow the traditions of the Prophet of Islam, Muhammad (pbuh), (Peace be upon him). A belief in the ‘unseen’ or supernatural world also forms part of Muslims’ belief system (Hossain, 2021). The unseen world includes angels and *jinn*, which is considered to be a spirit that can be good or evil and has the ability to cause harm in humans through possession (Badawi, 2017). Angels are perceived to be pure and sinless, while jinn are classified as either believers or disbelievers of God (Badawi, 2017). Jinn are thus not inherently evil. Belief in *sihr* (magic) is also included in discussions of the unseen as they are often linked to evil or disbelieving jinn. Lastly, Muslims believe in an afterlife, with this world as temporary, separated by a day of judgement where all actions will be evaluated (Abdullah and Nadvi, 2011).

Muslims in this study have a unique history. Cape Muslims are largely descendants of enslaved people brought from Asia and East Africa, ethnically named ‘Malay’, and later incorporated into the ‘colored’ category during Apartheid (Fataar and Esack, 2009; Vahed, 2021). Despite this incorporation, they maintained a distinct identity due to their religious beliefs, cultural traditions and sense of community (Fataar and Esack, 2009). The colored community is part of historically oppressed communities in South Africa (SA), who experienced marginalization and segregation (Netshivhambe, 2025). The Apartheid system of SA, rooted in racial segregation, enacted laws which divided the population socially, economically and politically from 1948 to the early 1990’s (Ayubi, 2023). The Cape Muslims thus hold a history that is complex, with experiences of slavery, colonialism, racial classifications and political struggle. The community expanded through slavery, intermarriage, conversions to Islam and cultural exchanges. They contest the fixed notion of ‘coloured’ and instead are constantly negotiating between multiple socially constructed identities. However, they retain their distinctness through their religion, Islam (Fataar and Esack, 2009; Vahed, 2021). Muslims’ lived experiences and resulting social constructions are thus influenced by their religion as well as their history as an oppressed people.

This study is situated within the epistemological framework of social constructionism, which views knowledge as socially constructed through shared belief systems and processes of meaning making. In other words, community experiences are not universal but instead shaped by their particular social interactions and cultural norms (Moradi and Yoder, 2001).

This study aimed to address the following question: what are the explanatory models of mental health conditions amongst Muslims in the Western Cape, South Africa (SA)?

## Methods

### Study overview

This research explored the explanatory models of mental health conditions amongst Muslims in the Western Cape, South Africa, from the perspectives of psychological service users and members of the general Muslim public. The study received ethical clearance from the Stellenbosch University Research Ethics Committee for Social, Behavioral, and Education Research (Project no. 23353). Guided by the principles of reflexive thematic analysis (RTA) as outlined by Braun and Clarke (2012), this qualitative, exploratory study aimed to uncover nuanced understandings of mental health within this context.

### Participant recruitment

Participants were recruited via MS’s social media networks (WhatsApp, n.d.; Instagram, n.d.), as well as through two online Muslim based self-development platforms (Muslims Connected, n.d.; Accidental Muslims, n.d.), utilizing purposive and snowball sampling strategies. Participants received no incentive to participate but were informed that their participation would contribute to expanding knowledge on Muslim mental health in South Africa. Individuals who self-identified as Muslim, were over the age of 18, spoke English or Afrikaans, and were either (a) treated by a mental health professional, or (b) from the general Muslim public were eligible to participate. A total of 21 participants who were treated by a mental health professional, and a total of 18 individuals from the general public were recruited.

### Data collection

Two data collection methods were utilized to accommodate the two types of participants. Semi-structured individual interviews were conducted with participants who had been treated by a mental health professional, which provided privacy for a personal, oft sensitive topic (Westland et al., 2025). Semi-structured focus group interviews were conducted with the general Muslim public, useful for group-based experiences and for increasing the richness of data (Ning et al., 2024; Tausch and Menold, 2016). The 18 participants were spread across five focus groups. A combination of in-person and online individual interviews (Microsoft Teams) were conducted according to participant preference. All focus group interviews were conducted online, anonymously, as per participants’ request. Individual interviews lasted between 35 and 75 min and focus group interviews lasted approximately 90 min. Both interviews covered beliefs, attitudes, perceptions and conceptualizations of mental health conditions, help-seeking behavior, and experiences in the community related to mental health conditions. Themes weighed equally for both participant groups except for discussions about lived experiences of mental health conditions. The lived experiences reflect narratives of psychological service users only. The interview schedules are attached (Appendix).

### Data analysis

In-person interviews were audio recorded and transcribed verbatim. Online and focus group interviews were video, and audio recorded, and automatically transcribed via the Microsoft Teams platform. All transcriptions were checked for accuracy. Note-taking was used throughout all phases of the analysis to track significant points in the data. As per Braun and Clarke’s (2012) RTA phases, firstly, familiarization of the data occurred by reviewing the video and audio recordings and re-reading the transcripts. Secondly, initial codes were generated inductively, and iteratively, using latent and semantic coding. ATLAS.ti. (Version 24 & 25), a computer assisted qualitative data analysis software program, was utilized. During the analysis phase, codes were checked regularly to ensure consistent and accurate coding. Codes were merged, collapsed or deleted where relevant. Upon completion of analysis, all code groups, codes and accompanying quotes were rechecked. Thirdly, initial themes were generated. Kleinman’s explanatory model framework and cultural formulation interview categories were used to help construct themes. Fourthly, themes were reviewed and refined to ensure accurate representation of the data. Themes were then named and defined to clarify scope and focus. Lastly, this paper integrates the analysis report with existing literature to provide contextual depth to the findings (Braun and Clarke, 2012; Joffe, 2012).

### Ethical considerations

Partcipants received electronic or hard copies of the university’s informed consent form, outlining the objectives of the study and expectations on participants. Informed consent forms were signed prior to the interviews and participant rights were verbally repeated at the start of each interview. Participants were informed that they had the right to withdraw from the study at any time. Details of free and low-cost psychological support services were shared with participants in the event of experiencing distress. Data were kept confidential using pseudonyms, and anonymity was preserved by presenting the data in themes, highlighting nuances, and using descriptive text (‘some’, ‘majority’, ‘a few’).

### Positionality

As Muslim practitioners, our positionalities are shaped by both shared faith identity and professional roles. MS, a Muslim female clinical psychologist and academic, conducted the interviews, drawing on insider status to facilitate rapport and open engagement. SAK, a Muslim counselling psychologist, supported the study through ongoing reflexive and methodological supervision. In line with reflexive thematic analysis, we acknowledge that knowledge production is shaped by researcher subjectivity. MS’s insider position was both a resource and a potential source of bias; therefore, reflexivity was embedded throughout the process. This included reflexive journaling and regular supervisory dialogue with SAK, to critically examine assumptions and power dynamics. We acknowledge the influence of our own positionality, experiences, and assumptions on the interpretation of meanings and the construction of thematic findings. Together, we aimed to ensure a transparent, reflexive engagement with the data while centering participants’ voices.

## Findings

### Participant characteristics

Participants totaled 39, with ages ranging between 18 and 60 years, and a mean age of 35 years. Eight men participated, three in the individual interviews and five in the focus groups. Women participants included 18 in the individual interviews and 13 in the focus groups. No other demographic information was collected.

### Themes and sub-themes

Seven key themes were constructed using Kleinman’s explanatory model framework. The themes are, (a) Naming and framing mental health conditions, (b) Beliefs about mental health conditions, (c) Attitudes toward mental health conditions, (d) Causes of mental health conditions, (e) Personal narratives of mental health conditions, (f) Challenges experienced with mental health conditions, and (g) Treating mental health conditions. For the purpose of this paper, mental health conditions, is used as an umbrella term to include all diagnosable psychological disorders. Findings suggest that no distinction is made between different psychological disorders in the community and all conditions are viewed the same due to lack of mental health literacy. An overview of themes and sub-themes is presented below, including a column depicting Kleinman’s explanatory model questions which informed theme construction. Quotes will be identified with ‘individual participant’ to indicate extraction from an individual interview, and ‘focus group’, for a focus group interview (see Table 1).

**Table 1:** Themes and sub-themes.

Theme	Question(s) from Kleinman’s cultural formulation interview	Sub-themes
Naming and framing mental health conditions	What do you call your problem? What name does it have?	Social labelling Cultural constructions of mental health
Beliefs about mental health conditions	How severe is it (sickness)? Will it have a short or long course?	Mental health beliefs Illness severity
Attitudes toward mental health conditions	What do you fear most about your sickness?	Attitudes about mental health conditions Attitudes toward individuals with mental health conditions
Causes of mental health conditions	What do you think caused your problem? Why do you think it started when it did?	Spiritual causes Biopsychosocial causes
Personal narratives of mental health conditions	What does your sickness do to you? How does it work?	Mental health experiences
Challenges experienced with mental health conditions	What are the chief problems that your sickness has caused for you?	Cultural responses to mental health conditions Lack of engagement amongst religious leaders
Treating mental health conditions	What kind of treatment do you think you should receive? What are the most important results you hope to receive from treatment?	Cultural beliefs about treatment Recommendations

### Naming and framing mental health conditions

Participants reported on the various ways in which mental health conditions were spoken about in the Muslim community, noting terminology and discourses used. This theme includes (a) social labelling and (b) cultural constructions of mental health.

#### Social labelling

Participants reported descriptors for any behavior considered to be indicative of mental health difficulties. These included terms like “emotional pain” (focus group 1), “emotional suffering,” “heartache” (focus group 2), “mental breakdown” (individual participant 4) and “nervous breakdown” (individual participant 6). However, the majority of participants highlighted a common practice of labelling individuals perceived to be experiencing mental health difficulties. The labels used were often negative, “she’s [cognitively] slow,” “either you are normal, or you are not” (focus group 1), and derogatory “. It’s also derogatory, but there was *taatie* (crazy)” (focus group 4), “the social outcasts were called *malletjies* (the mad ones)” (focus group 5). Terms like *malletjie* and *taatie* were disparaging towards people with mental health conditions, and the term *taatie* originated from the word ‘retarded’. Other terms used were “unstable” (individual participant 6), and “psycho, crazy, off a rocket…not so *lekker* (good) up there” (individual participant 10).

Participants emphasized the negative associations with these labels, as they indicated limited functionality, “that person can no longer function” (individual participant 9). They also highlighted that these labels influenced their own experiences with mental health difficulties, “I think that is a big reason why I tend to…reject mental illness…because it is viewed negatively” (individual participant 11).

#### Cultural constructions of mental health

These labels also influenced the way mental health conditions were perceived and spoken about. Ensuing cultural rhetoric about mental health conditions contributed to how mental health was experienced in the Muslim community. Participants reported that mental health conditions are often perceived as an experience that does not affect Muslims, “as a Muslim, people expect you to not get depression or mental illness…because we have *duas* (supplication) and *solaah* (prayer) and your connection with Allah” (individual participant 17). Furthermore, the community believed it existed only within other communities and societies, “a made-up Western concept” (individual participant 15), “depression *is wit mense se ding* (depression is a white people thing)” (individual participant 7). These constructions influenced how individuals with mental health difficulties were treated. Individuals were perceived as “overly sensitive” (individual participant 6), “being unnecessary” (individual participant 1), called lazy, “*jy wil net daar lê en niks doen nie* (you just want to lay there and do nothing), then you will say but you are not feeling well, ‘daars niks verkeerd met jou nie (there is nothing wrong with you), you are lazy” (individual participant 13), and accused of seeking attention:

*When I was growing up, if you felt hurt or sad, it’s just ‘hy is stout’ (he is naughty), or ‘hy soek vi aandag’ (he is looking for attention), that’s normally the type of answers that you get. Or ‘los sy sal reg kom’ (leave it, she will come right). It was like, there wasn’t such a thing as depression and feeling sad* (individual participant 18).

Discourses in response to individuals experiencing mental health difficulties were dismissive, “hou op misluk wies (stop being moody)” (individual participant 7), and they were instructed to simply move on, “people need to pull themselves together…*ruk them reg*” (pull yourself together) (individual participant 1) and “roll with the punches” (individual participant 15). Lastly, experiencing mental health difficulties also meant that individuals could not be trusted, “*dai klong moet ek dophou* (I must watch that boy), *hy is mal weet jy* (he’s mad, you know)” (individual participant 7).

Cultural constructions of mental health conditions were predominantly negative and exacerbated the experience of mental health difficulties.

### Beliefs about mental health conditions

Participants shared beliefs associated with mental health conditions in the Muslim community. The beliefs comprise of, (a) mental health beliefs, and (b) illness severity.

#### Mental health beliefs

Beliefs about mental health conditions in the Muslim community varied. Participants reported that in the community, many believed that mental health conditions were not an actual health condition. Mental health difficulties were also commonly believed to be part of life, to be endured, or interpreted through a religious lens. For example, participants shared, “When I was younger, I never knew that mental illness existed” (focus group 5), and “I feel like it’s not a real illness” (individual participant 20). Interestingly, participants also perceived life challenges not as experiences to be processed or addressed, but rather as conditions to be endured—something they could not afford the luxury of engaging with more reflectively, “We just endure the hardships” (focus group 4), “Life needs to go on. I am a mother of *[many], it cannot be all about me” (individual participant 5), “we always just knuckle down and do what needs to be done” (individual participant 7). Lastly, viewed through a religious lens, participants believed that all hardships come from God, with a purpose to strengthen their spiritual connection. About this, participants shared:

*“You need to suffer in order to be good” (individual participant 8),*

*“Our deen (religion) is our basis” (individual participant 9),*

*“So, only because of Allah, that’s why I experience pain and suffering…there’s a greater purpose” (focus group 2),*

*“Opportunity to strengthen my relationship with Allah” (focus group 1).*

#### Illness severity

Participants’ beliefs about illness severity were broad. They reflected on their initial understanding of mental health difficulties, informed by religious and cultural beliefs. Firstly, mental health conditions were generally viewed in an extreme form, “someone that has a psychiatric disorder requires being locked up in a straitjacket” (individual participant 10). Participants viewed mental health conditions as an invisible difficulty, “the person who suffers from it is going through a disorder that is not physical. It’s something we cannot see” (focus group 2), and an experience that can affect physical health as well, “I do believe that mental health impacts also physical health and vice versa” (individual participant 8). They described mental health conditions as behaviors that were outside societal norms, “debilitated to an extent that you exhibit irrational behaviors, that is contrary to the norm that you find in societies” (individual participant 15), and it brought on mental, emotional and even spiritual difficulties, “it could be emotional; it could be mental” (focus group 2), “from a Muslim perspective, your mental health and your physical health links to your spiritual health” (individual participant 3). Participants further described mental health conditions as something that affects how individuals engage in their lives, “a mental disorder to me is something that becomes a barrier between you and fulfilling a task” (individual participant 1), and “somebody that is not fully in control of themselves” (individual participant 7). Lastly, participants believed that mental health difficulties have always been recognized by Islam, “…I think there’s even narrations of the prophet struggling with his own challenges. So, it’s always been there, and Islam has always had the remedy for it” (individual participant 12). This is an interesting point considering the amount of stigma in the Muslim community reported by participants.

Participants’ beliefs about mental health conditions were varied, informed by community assumptions which reflected little compassion for those experiencing mental health difficulties.

### Attitudes toward mental health conditions

Attitudes toward mental health conditions were twofold. The sub-themes include (a) Attitudes about mental health conditions, and (b) Attitudes toward individuals with mental health conditions.

#### Attitudes about mental health conditions

Participants reported that mental health conditions were considered taboo in the community and thus perceived negatively, “it’s something that is very much [a] taboo subject” (individual participant 10), “it’s only negative” (focus group 2), “it’s not always well received” (individual participant 8). As a result, mental health conditions are perceived as something shameful and stigmatized, “Mental health is so stigmatized I feel in the Muslim community, that it becomes a matter of shame” (focus group 2). These attitudes were informed by external pressure to be perceived as mentally well, “it’s all about status…what the family thinks…what other people think, instead of focusing on trying to help and guide that person that is suffering” (focus group 3), “if you say that you are struggling mentally…there’s a stigma saying you are not good enough” (individual participant 1). Mental health conditions were thus not spoken about, “it’s not an open conversation” (focus group 1), “because these things do get swept under the carpet or it’s just brushed off” (focus group 2), and individuals were explicitly prevented from discussing their mental health conditions with anyone, “the person had seen me behave differently…I wanted to explain myself, but I wasn’t allowed to” (individual participant 1). The negative attitude towards mental health conditions overburdened individuals experiencing it, “that’s the only negative thing” (individual participant 21), and resulted in “fear” for those experiencing mental health difficulties and “disappointment” by their families (focus group 3).

#### Attitudes toward individuals with mental health conditions

The attitudes toward mental health conditions influenced the way individuals with mental health conditions were treated. Participants reported that individuals were often feared, because they were perceived to be unpredictable and strange, “We normally kept our distance because they are not safe. They’re not secure and anything can trigger them. They could have violent outbursts and so forth…” (individual participant 14). Individuals were subsequently shunned by the community and treated like outcasts, “…they chose to be offensive and block her out or ignore her” (focus group 1), “…move away, be scared of them, shun them…” (individual participant 1). Individuals were typically treated with a lack of empathy:

*Hy is taatie, daar is iets verkeerd moet hom (He is crazy, there is something wrong with him). And that’s it, there’s no further discussion…there is no empathy, we lack empathy when we speak about people with mental illness because, ‘dit gebeur nie met os nie’ (it does not happen to us)”* (individual participant 7).

This type of treatment draws attention to the influence of culture on attitudes, as this kind of behavior can be seen as a violation of Islamic ethics, a system that promotes justice and fairness, kindness, compassion and empathy towards others (Ebrahimi and Yusoff, 2017; Ali et al., 2024).

The lack of empathy contributed to individuals with mental health conditions being mocked and belittled, “I used to laugh at these people because other people laughed at them” (focus group 5), “her own siblings treated [her] like she was nothing” (focus group 1). Participants believed that individuals were treated this way because there was a lack of understanding in the community about mental health in general, “definitely lack of awareness, lack of understanding, understanding what it is, number one, and understanding number two, the extent of what it can cause for the person who is going through it” (individual participant 20). They also reported limited opportunities for understanding mental health conditions, “there wasn’t much education around it” (individual participant 14), and often highlighted the lack of understanding amongst older individuals in the community, “people are talking about it more, but I guess it’s our older community. Our older folk that struggled to grasp what it is” (individual participant 9). Many Muslims experienced a lack of support as a result, “I know a lot of people that go through this [no support] in the Muslim community” (individual participant 21), contributing to serious mental health risks for them:

*It’s all synonymous with how we as a people treat it. So, we do not go and find out what is the actual problem that the person [has], we do not know, is it physical, we do not know what is actually wrong and we just leave it and that person just suffers or gets committed or commit suicide* (individual participant 7).

Attitudes toward mental health conditions were often negative and contributed to how community members responded to individuals with mental health conditions. These negative attitudes reinforced the lack of understanding about mental health conditions and Muslims with mental health conditions were often left isolated and without support.

### Causes of mental health conditions

Causes of mental health conditions fell into two categories. These two categories were informed by participants’ understanding growing up in the Muslim community, and secondly, their understanding after they were exposed to mental health conditions and educated about it. The two overarching causes were: (a) spiritual causes, and (b) biopsychosocial causes.

#### Spiritual causes

Participants reported that mental health conditions were perceived to result from a lack of religiosity and the individual was subsequently being punished by God, “it was just, you are either not a good Muslim or your spiritual level with *Allah* (God) is not right if you have a mental illness” (individual participant 8), “I think that’s what people think it is. They think that if I’m sitting with the issue like this, *Allah* is punishing me because I did not do ABC” (individual participant 10). Poor religiosity as a causal factor was prominent, “people in the community would say your *emaan* (faith) is weak” (focus group 3). Interestingly, Muslims who were experiencing mental health conditions sometimes viewed the experience as a spiritual test from God, “*Allah* SWT gives us our own test, so I did [think of it like that] as a coping mechanism, for myself. I just realized that this is my test, and I must pass it” (individual participant 1). The test was not perceived as a punishment, but rather to get closer to God, “*Allah* is showing me this, so how can I move past it or how can I become better to get a step closer” (individual participant 8). For some of them, the experience resulted in a positive outcome – closeness to God. For others, it caused them to question their faith, “what’s happening to me Allah, why [are] you doing this to me? Am I not a good enough Muslim?” (individual participant 2), “So, there was a lot of confusion…understanding challenges of my own was often times – is it related to the fact that I’m not a good Muslim?” (individual participant 6).

Lastly, one of the most perceived causes for mental health conditions amongst the Muslim community was the idea that it was caused by some kind of supernatural experience. Supernatural experiences included jinn possession:

*I do not even know how to explain it, but it would be almost like, they do not use the word possessed, but it’s like you are* gepla *(affected) with the jinn. You know, there’s a spirit that’s causing these things. I suppose, like you are mildly possessed* (focus group 2).

Additional supernatural explanations included being influenced by *shaytaan* (devil), “I heard of *shaytaan*, maybe one cause of mental illness, because if you are listening to him and giving in to your desires, [that] could put you in a really dark spot” (focus group 3). Black magic, which is commonly defined in academic literature as the intentional use of supernatural or magical practices to cause harm, misfortune, or negative outcomes to others and is often conceptually equated with witchcraft (Moro, 2018), was also noted as an explanation, “coming from a Muslim background, a lot of the time(s), people would assume that mental illness is directly linked to black magic” (focus group 4), as well as *evil eye.* Evil eye is a curse believed to be cast by a malevolent glare, usually directed towards a person who is unaware of it. Many cultures believe in the evil eye as a reason for bad luck, illness and injury (Daher-Nashif et al., 2020, p. 2061). Although nafs does not directly translate to evil eye, the cultural use here makes reference to it):

*Peoples’* nafs *on you makes you get sick, because when my mom reflects back on her [inpatient] experience, she always used to say: ‘*die mense het gesê ek het groot nafs op my*’ (the people said I had great envy on me)* (individual participant 4).

Finally, witchcraft was also noted as a possible explanation for mental health conditions experienced, “because he started speaking to himself and you know, they obviously blamed witchcraft for the reason he is the way he is” (individual participant 18). Participants reported that these supernatural explanations were often an immediate response to the experience, “automatically it was that I was possessed and there was something wrong with me and it’s so negative” (individual participant 11). Mental health conditions were also thought of as self-inflicted, “you brought it upon yourself” (individual participant 16). Belief in the supernatural world is part of Muslims’ faith and includes *jinn* (spirit), angels, *shaytaan* (devil), *sihr* (black magic), the *barzakh*, a place where the souls of the departed reside until the day of resurrection (Harahap et al., 2024), and the afterlife (Khan et al., 2024; Fawad et al., 2019). However, in this context, the supernatural is conceptualized as a culturally embedded explanation for mental illness, wherein supernatural beings are believed to affect an individual’s psychological state (Lim et al., 2018).

#### Biopsychosocial causes

Participants acknowledged that an interplay of different factors may contribute to the development of mental health conditions, “I do believe that multiple factors affect people’s mental health” (individual participant 7). These factors included medical causes or genetics, “there’s chemical imbalance, which is a physical thing which can affect somebody’s mental state…thyroids that are not normal” (individual participant 7), and “now I know the neurological function behind mental well-being” (individual participant 10), “some mental disorders can be hereditary” (individual participant 15). Secondly, participants noted various psychosocial causes, “I think largely trauma…generational trauma” (individual participant 3), “there could be significant life events, death, divorce…” (focus group 2), and “I have an aunt who was involved in an accident, and it affected her mental well-being” (focus group 2). They also reported that “the environment” (individual participant 3), including social media can affect ones mental health, “I even believe that being active on social media for copious amounts of time and indulging in certain topics on social media can mess with your mind and start a fight within your mind” (focus group 5). An inability to cope with challenges, “your ability to maybe just cope with certain situations, you cannot, you really cannot” (individual participant 2), substance use, “drug induced” (individual participant 18), and our immediate environment was also noted as potential causes for mental health conditions, “and also your circumstances, the environment that you are in” (individual participant 19). It was evident that most participants embraced the biopsychosocial model of understanding mental health conditions, “Mental health struggles have definitely been linked to chemical levels, imbalances [in the brain] … Some people have a predisposition just genetically…then the order of their life will determine whether it is exacerbated by stressful life events” (individual participant 6).

Participants reported that initially, their explanations for mental health conditions were informed by their community, which included lack of religiosity and supernatural explanations. However, as they were exposed to mental health conditions, especially those who experienced it themselves, their mental health literacy increased and they developed a more informed, and accurate understanding of explanations for mental health conditions.

### Personal narratives of mental health conditions

Participants reflected on their experiences with mental health conditions and how it affected them in different ways. This theme was informed by the narratives of psychological service users only.

#### Mental health experiences

For some participants, the mental health difficulties affected them emotionally, “I just feel dead most of the time…it’s difficult now for me to like put my emotions out there ‘cause I feel like I’m always on guard (individual participant 5), to the point where they felt like they could not function at all, “I thought I am like officially losing it, like, is this the point at which I am officially, you know, not healthy anymore and not functional” (individual participant 6). Although participants never mentioned suicidality explicitly, they reported experiences which indicate the symptom was present, “I felt a lack of wanting to live then. I never saw any joy, I was angry, I was always upset” (individual participant 8). These experiences also affected participants’ confidence in their abilities, “I felt like I was dumb because why was I struggling with some things that were so simple” (individual participant 3), “it was a knock on my ego, on my inner self” (individual participant 7). The experience often left them confused because sometimes they felt physically able, but were not able to carry out tasks, “physically I feel fine, I can walk a mile, but mentally I cannot carry this heavy thought man” (individual participant 13). On the other hand, some experienced physical symptoms which alerted them, “my heart rate wasn’t right. I had high blood pressure. I just a lot of things. My body just shut down completely. Everything was just not in balance” (individual participant 19). Some symptoms were prominent and eventually recognized by others:

“*I left my work. I stayed at home. I did not wanna eat. I did not wanna go out. I was just like in my room all the time. You do not do the things that you must do on a daily basis. I just cut myself off until somebody noticed what was happening to me”* (individual participant 17).

Participants’ experiences of mental health difficulties often left them overwhelmed and affected their confidence in themselves. It was a completely new experience to navigate, as they had no prior exposure or knowledge about it.

### Challenges experienced with mental health conditions

Participants from both stakeholder groups commented on how perceptions of mental health conditions in the Muslim community contributed to the challenges experienced. They noted that these negative views were problematic as they further fueled stigma associated with mental health conditions leading to poor health-seeking behavior, and isolated individuals living with mental health conditions. Challenges included, (a) cultural responses to mental health conditions, and (b) lack of engagement amongst religious leaders.

#### Cultural responses to mental health conditions

Participants reported that because mental health conditions were perceived to be non-existent or unimportant, mental health difficulties were not acknowledged, and responses were often indifferent:

*You know the response to everything in the older aunties circles when somebody is dealing with the* janazahs *or funerals, you know, people are at the point where they are going to break down so emotionally, I’ve seen it so many times. Their response to everything is to get someone a glass of sugar water.* (Focus group 2).

The norm in the community was to struggle and endure, and mental health difficulties were viewed as part of ‘life problems’ to be survived like everything else:

*Even when it comes to like trying to help my mother to go for counselling because she is depressed; she gets so offended that you think that she needs help mentally. She keeps like “no, I’m fine, there’s nothing wrong with me, I’m fine,” so there is just that closed off. There is that stigma I think for them, for the older generation… For them they just think it’s normal, that’s how life is. They’re in that kind of survival of some sort… I mean the wars they fought, the people they lost, the trauma, there’s a lot of things that you do not just ignore. I think that in our stories we kind of romanticise this thing that we fought, and we were brave, and we did not have to deal with anything afterwards. Whereas we do*. (Individual participant 8).

Despite attempts to build mental health literacy in the community, resistance remained, “I just feel that there are people trying to talk about it, but there’s also people pushing it back” (focus group 5). It is noteworthy that the resistance to understanding or merely engaging with mental health was influenced by both the culture of the society, as well as their religious views, “I do think that religion plays a role in limiting, limiting people, preventing, I do not want to say preventing but I suppose restricting that people do go and seek professional help” (focus group 2). Participants reported that in the Muslim community, the sole focus was on religiosity as a determinant for a healthy life, “I think that’s related to the religion thing where people insist on, if I’m making my *solaah* (prayer), if I’m doing everything I need to do, I should be fine” (individual participant 10). Thus, religious action was viewed as preventative; if you live a good Muslim life, you will remain mentally healthy.

#### Lack of engagement amongst religious leaders

The majority of participants shared that the lack of engagement by religious leaders also contributed to the inaccurate perceptions of mental health in the Muslim community, “up until today, religious leaders are not talking about it. They do not see it; they only believe that you must have faith. You must have *sabr* (patience)” (individual participant 14). Some participants experienced a slight shift in religious leaders’ understanding of mental health, “some of the scholars that I have interacted with have said that if you are at that point where you literally cannot get up, you do not have the motivation, then you need to go find professional help” (individual participant 12). It is clear though, much more is needed in the community to dispel the misunderstanding and stigma around mental health conditions, as well highlight the importance of addressing the experiences of historical trauma brought on by apartheid in the community:

*So, number one, religious leaders need to actually acknowledge that this is a growing concern in the Muslim community. Number two, they need to talk about it and talk about it on the radio stations to get the older generation to understand why, what, and how, and also remember they have got a block on their mind as well. They were brought up in this way not to talk about it. They use negative connotations mostly in the old generation. So, you need to break down that initial barrier ‘cause they also have psychological trauma that they are still busy dealing with. Back in the day it was apartheid issues and all these things that added to this. So, they were carrying the sins of their father, so to speak, for a long time*. (individual participant 14).

Participants reported on the challenges they experienced in the Muslim community. They highlighted the cultural responses to mental health conditions and how it facilitated negative experiences for them within the community. Lastly, they reported that the absence of religious leaders in creating awareness about mental health conditions contributed to the stigmatized view.

### Treatment of mental health conditions

Participants reported various treatment and health-seeking behaviors within the Muslim community. The type of treatment sought was influenced by how mental health conditions were constructed. They also shared recommendations for better health-seeking. Sub-themes include a) cultural beliefs about treatment, and b) recommendations.

#### Cultural beliefs about treatment

The belief that mental health conditions were due to poor religiosity prompted treatment that would address the lack of faith. Treatment imposed thus included some kind of religious action like thikr, the act of remembering Allah by the means of recitation and reflection. It involves the repetition of the name of Allah to nurture a deeper spiritual connection with Him (Daniyarti et al., 2023):

*If you just make* dua *(supplication) and make* solaah *(prayer) and recite certain things, it will go away* (individual participant 10).

*People will tell you “No man, make thikr,* sit op jou muslah *(sit on your prayer mat)* (individual participant 13).

In instances where supernatural causes were suspected *Ruqyah*, an exorcism, was recommended. *Ruqyah* refers to the recitation of verses from the Qur’an for spiritual healing. It is regarded as a treatment used to heal against the effects of evil eye, illness, poisoning, or jinn-possession (Al Salam Islamic Center, 2023):

*I think when it comes to the, the* jinn *and those types of things, “da is die* boetajie*” (there is an uncle), there is always the “*boetajie*” (uncle) that you must go to. “Hy gaan jou uit mantra” (he will exorcise you), that’s the first thing* (individual participant 7).

*Let us get the Imam (cleric), batcha (recite), thikr (supplication), myang stokkies (incense sticks), lots of different things that, depending on the Imam again, where they come from and what they try. But most of the time it’s like Quran recitations, it’s just basically getting the person with them, being with them and reciting Quran. Some people do different things at different times* (focus group 5).

There were also cultural responses to experiences of mental health difficulties:

“*Instead of setting them down, instead of letting them have a conversation with you about how they are feeling, about what it means to them, that they lost their mother, about what do they need to be OK, or letting them not be OK in that moment. Letting them grieve. Our response is, give them a glass of sugar water*. (focus group 2).

Participants described receiving sugar water during periods of emotional distress, which may reflect local folk-healing or home-care practices embedded within broader cultural traditions of responding to suffering and illness (Shange and Ross, 2022; Sorsdahl et al., 2010). However, while this practice may demonstrate care in a moment of need, participants lamented the lack of further engagement with their distress and perceived a tendency within the community to move beyond such experiences without sustained acknowledgement or support.

Although minimal, several participants from the focus groups noted psychological treatment approaches. These included psychotherapy and psychopharmacological management, “I think it can just be treated with medication, therapy and counselling” (focus group 5), “So, for me, it’s around a lot, in terms of just processing what happened and how to actually deal with those feelings related to that circumstances” (focus group 2).

Formal health-seeking was not the first option for many in the Muslim community. However, individuals with mental health conditions were able to seek appropriate treatment despite being instructed only to engage in religious activities or seek an *Imam’s* intervention. Many participants from the focus group reported similar treatment trends from their own families, but indicated that mental health awareness, including participating in the current study, made them more open to seeking formal treatment should they ever require it:

*Personally, yes, I would see a psychologist if I need to. Before I maybe would not, but my perspective changed now on this whole conversation that people actually do need help* (focus group 1).

*I would actually, and to be honest, it’s only because my perspective that I have now and this year has changed since last year and the previous years. And that’s only because this year and once I started working, I started speaking more to people. And I’ve heard of more Muslims that have gone to therapy and how it is a normal thing and how they have dealt with it and now they are doing better* (focus group 3).

Interestingly, there were participants who reported that they would not seek formal treatment regardless of how they viewed mental health conditions. Highlighted in the quote below is the anxiety around disclosure and reliance on a higher power when in distress. This is perhaps also an indication of the broader Muslim community, and how similar beliefs continue to persist:

*I do not. I honestly do not think I would want to go see a professional or seek professional help. Just because of the reason, firstly actually speak to your parents about you are suffering a mental illness or you are feeling this kind of way, which is something I feel I’m not ready for. And also, with my beliefs on speaking to Allah about your problems, and yes, sometimes we feel that when our emaan is not strong, Allah might not accept or we do not see the solutions that Allah is putting out to us, and sometimes I feel more comfortable speaking to a friend* (focus group 3).

#### Recommendations

Participants highlighted the need for more conversations about mental health in the Muslim community. They believed that creating awareness, and including Islamic scholars with these conversations will help de-stigmatize mental health, build mental health literacy and promote appropriate health-seeking behavior:

*I think this is the type of conversations that our Islamic scholars need to, I suppose, also engage in because I think [she] touched on the fact that they are not equipped to support when it comes to mental conditions. And I think if the Islamic scholars can get on board a little bit with that and then just combine that with the Islamic teachings, I think that will just really benefit the Muslim communities.* (Focus group 2).

Finally, while they believed that psychoeducation was important, “I feel like lots of psycho education [is needed] for Muslims, because sometimes it’s swept under the carpet or it’s just not recognized as a proper illness” (individual participant 20), they also acknowledged the responsibility within the Muslim community to begin changing the narrative:

*I guess that’s our roles, and our responsibility is to speak to our elders. To also educate them, to make them realise that this is real, it does not just happen to people who do not have “emaan” (faith). So, I guess it’s on us. And also, on us to instill in our kids that you know it’s not something that we just leave and shove under the carpet. It’s something we need to deal with*. (Individual participant 9).

Participants reported differing treatment seeking behaviors in the Muslim community, as well as ideas to aid appropriate health-seeking for mental health conditions in the Muslim community.

## Discussion

This study explored the explanatory models of mental health conditions amongst the Muslim community in Cape Town, South Africa. Although not included in the shared quotes, participants welcomed the study as a platform to finally share their experiences. However, they perceived authors as *brave* for ‘touching something nobody wants to touch’ (focus group 1). Their response to the study provides an interesting departure point in understanding mental health in the Muslim community.

### Explanatory models of mental health conditions

Findings revealed several significant insights. Firstly, the study highlighted both religious and cultural explanations for psychological distress. These informed attitudes and beliefs about mental health conditions, which were mostly negative, and consequently informed attitudes towards mental health conditions and individuals experiencing mental health conditions. While some participants had evolved in their explanations of mental health conditions, majority of participants indicated continued lack of understanding in the broader community. Participants highlighted the lack of mental health literacy, which contributed to the way mental health conditions were explained. The lack of mental health literacy was a dominant theme with participants proposing psychoeducation as a simple yet effective resource that could be used to combat the negative perceptions of mental health conditions and help encourage more formal help seeking.

### Culture and mental health

Notably, participants shared experiences of ostracization and derogatory terminology due to experiencing mental health conditions. This theme was raised repeatedly by individuals who had been treated by mental health professionals. The ostracization left them feeling isolated, with little to no family or community support, and this experience exacerbated their difficulties. The way participants were treated is in direct contrast to Islamic ethics, which promote kindness, compassion and justice (Ebrahimi and Yusoff, 2017). Participants attribute the treatment to the continued stigma around mental health conditions. In fact, the stigma in the community was often described as more challenging than the experience of mental health conditions itself. We noted a pattern similar to what has been found previously and in other groups: culture is implicated in conceptualizations of mental health conditions (Hassim and Wagner, 2013); these conceptualizations include misconceptions which create stigma (Egbe et al., 2014); help-seeking is delayed (Sikrweqe et al., 2024); and this contributes to poor mental health outcomes (Elshamy et al., 2023). This contrasting experience was described by participants as a cultural rather than religious response, as the community had a particular manner of responding to mental health experiences. Participants’ distinction between cultural and religious responses is consistent with cultural psychiatry perspectives which view culture as a product of broader social, political, and historical processes that shape community understandings of illness and appropriate responses to distress (Kirmayer, 2012).

Historical trauma was often reported as embedded in peoples lived experiences, contributing to how they made meaning of mental health conditions. Although not explicitly stated, South Africa’s history of racism and marginalization was often alluded to as the context in which the community developed their ‘survival mechanisms’. Kleintjies and Schneider (2023) provide a detailed account of the history and politics of mental health policy and care in South Africa. Here we draw attention to two points mentioned by Kleintjies and Schneider (2023), (1) the effect of the apartheid system on the wellbeing of South Africans at the time, and (2) the intergenerational psychological trauma created by the experience of land dispossession, segregation and oppression. These experiences have created the survival mechanisms we find in communities, even currently, including in the Muslim community. During apartheid, health services, including mental health services were segregated by race (Mkhize and Kometsi, 2008), with grossly unequal distribution of services prioritizing the white minority who were in power at the time. Therefore, many in the Muslim community have not been exposed to psychological services, which is perhaps why many believe that mental health conditions is a ‘*white people’* experience.

In the case of Cape Muslims in particular, Islam’s roots in slavery (Baderoon, 2018), further demonstrate experiences shaped by volatile sociopolitical environments. This history in combination with social realities influence how meanings are made and instead of seeking care, mental health experiences are survived and endured.

### Religion and mental health

Social constructionism theorizes that knowledge is contextual, and meanings are created through social discourses (Moradi and Yoder, 2001). This was evident in the shared experiences and understanding about religion and mental health. Regarding religion, study participants highlighted common beliefs around the legitimacy of mental health conditions, where the broader community did not perceive mental health conditions as a valid health condition. Further, some noted a common belief that Muslims who remain adherent to Islamic traditions, *should not* develop mental health conditions. Engaging in compulsory Islamic rituals was perceived to be sufficient in maintaining a healthy mind and body. Thus, any experience of mental health conditions was framed within a lack of, or poor religiosity, requiring an increase of religious based rituals and activities (Weatherhead and Daiches, 2010; Fekih-Romdhane et al., 2023; Moodley et al., 2018; Alqasir and Ohtsuka, 2024; Elshamy et al., 2023). This imposed assumption often left individuals with mental health conditions feeling judged, leading to experiences of social isolation and seeking treatment under duress, in secret.

In addition, a second common explanation for mental health conditions was the experience of supernatural forces as highlighted in previous findings, amongst similar and other groups (Dein, 2020; Subu et al., 2022; Alqasir and Ohtsuka, 2024; Ayub, 2021; Saged et al., 2020; Kpobi and Swartz, 2019). These included spirit possession, devil influence, black magic, and evil eye or envy. In the current study, when these were considered as a primary explanation, treatment sought included input from religious leaders only, disregarding any kind of formal treatment. Participants noted these explanations were due to a lack of mental health literacy in the Muslim community, perpetuated by a complete lack of discussion on this topic. Some participants also noted that the lack of discussion amongst Muslim clerics as a trustworthy source, further perpetuated the misinformation about mental health conditions. It is important to add that Muslims acknowledged the existence of supernatural beings, believed in influences from the supernatural world, but felt that in this instance, the supernatural explanations were inaccurate and created a barrier to formal mental healthcare.

### Extent to which EM contributes to health seeking

A recurring theme in the findings is how explanatory models influence the health-seeking behavior. Spiritual treatment was often the first option, with formal health-seeking delayed or not sought at all. There appeared to be a constant juxtaposition in their experiences, and a continuous tension between culture, religion and new knowledge. Despite learning about mental health conditions, a few participants still indicated they would *not* seek formal treatment. A few participants shared that even though they experienced mental health conditions, they continued to ostracize others with mental health conditions. Participants highlighted the constant conflation of culture and religion and how that informs the explanatory models of mental health conditions, which influences health-seeking. Lastly, many believed that deeper Islamic knowledge would influence Muslims’ understanding of mental health conditions and would make them more accepting of it as Islam promotes holistic wellbeing, which includes mental health (Awaad et al., 2019; Laksana and Hisham, 2025).

## Conclusion

This study explored the explanatory models of mental health conditions amongst Muslims in Cape Town, SA. Findings highlight the intersectional nature of mental health experiences of South African Muslims. Cultural and religious conceptualizations of distress, low mental health literacy and stigma in the community and historical experiences of dispossession and racial segregation were all highlighted. A conflation of culture and religion, masculinity and mental health, and ostracization and isolation of those experiencing mental health conditions was emphazised as well. Lastly, and importantly, explantory models of mental health conditions are implicated in health-seeking behavior.

### Clinical implications

Understanding the explanatory models of mental health conditions among Muslims in the Western Cape carries important clinical implications, particularly for enhancing culturally responsive assessment and intervention. Clinicians who appreciate clients’ spiritual, sociocultural, and relational understandings of distress are better positioned to build therapeutic alliances grounded in cultural humility and respect. Such insight supports more accurate case formulation, reduces the risk of diagnostic bias, and enables the integration of faith-aligned coping strategies, such as religious practices, community support, and collaboration with trusted religious leaders, where appropriate and desired by clients. These findings also highlight the need for tailored psychoeducation to address stigma, misconceptions, and moral interpretations of mental health conditions, thereby improving help-seeking pathways and reducing delays in accessing care. The findings further suggest that explanatory models extend beyond beliefs about the causes of mental health conditions and are embedded within broader historical, cultural, religious, and social contexts. Factors such as apartheid legacies, migration histories, intergenerational trauma, community norms, family expectations, religious worldviews, and stigma shape how distress is understood and experienced. These meaning systems, in turn, influence emotional responses, help-seeking behavior, and engagement with mental healthcare. Rather than functioning solely as causal explanations, explanatory models provide broader frameworks through which distress is interpreted, negotiated, and managed. Finally, the study underscores the importance of ongoing clinician training in cultural humility and Islamic perspectives on mental health to ensure that services remain accessible, acceptable, and contextually attuned to the needs of Muslim communities in South Africa.

### Limitations

This study was conducted among Muslims residing in Cape Town, a community shaped by a distinctive historical, cultural, and sociopolitical context. The findings should therefore be interpreted within this setting. While transferability to other Muslim communities in South Africa is possible, caution is warranted given the diversity of South African Muslim populations with respect to race, migration histories, language, and community identities (Vahed, 2021). Furthermore, research on Islamic conceptualizations of illness in South Africa suggests that understandings of mental distress are shaped by local cultural and religious contexts rather than reflecting a singular Muslim perspective (Laher, 2014; Moodley et al., 2018). As such, further research in other South African regions would be valuable in exploring how explanatory models may converge or differ across Muslim communities.

In addition, many participants had personal experience of mental health difficulties or prior exposure to mental health services. Their accounts therefore reflect perspectives informed by engagement with mental health concerns and may differ from those of community members with limited exposure to mental health information or services.

Lastly, participants were recruited primarily through online and social media platforms. Consequently, participation was restricted to individuals with internet access and engagement with digital networks. Given persistent digital inequalities in South Africa, online recruitment may have underrepresented individuals with fewer digital resources or lower levels of online engagement, thereby limiting the breadth of perspectives captured (Partridge et al., 2024).

## Data Availability

The original contributions presented in the study are included in the article/Supplementary material, further inquiries can be directed to the corresponding author.
