# Supplementary material for: Between culture and religion: explanatory models of mental health conditions amongst Muslims in Cape Town, South Africa
Source: Front Psychol. 2026 Sep 25;17:1910057. doi: 10.3389/fpsyg.2026.1910057 (PMC13649462; doi:10.3389/fpsyg.2026.1910057)
Supplement: Supplementary file 1 [file Data_Sheet_1.DOCX]

**Appendix**

**Focus group schedule**

***General Muslim public***

1. Name
2. Age
3. Gender
4. Can you tell me how you make sense of pain /suffering that is not physical?
5. Is there a name that you have for this kind of pain?
6. Have you ever been a part of a discussion in your community about this kind of pain/suffering?
7. What do you understand by the term mad/malletjie? Do you know any other terms like that?
8. Have you ever heard of the term mental disorder?
9. What do you understand by the term mental disorder?
10. Have you ever been exposed to someone with a mental disorder?
11. What do you think is the cause of illnesses that are not physical (mental disorders)?
12. Do you think these illnesses can have more than one cause?
13. What is your understanding of this kind of illness from an Islamic perspective?
14. What is your understanding of jinn (spirits), shayateen (devils), Marrid (demons) and bhut (evil spirits) regarding this kind of illness?
15. Would you consider seeking professional help (from a doctor) if you suffered from an illness that is not physical?
16. Does religion play a role in decisions to seek professional treatment?
17. Is there anything that you would like to tell me about your understanding of

mental disorders that I haven’t asked you?
